# The impact of EGFR mutation status and single brain metastasis on the survival of non-small-cell lung cancer patients with brain metastases

**DOI:** 10.1093/noajnl/vdaa064

**Published:** 2020-05-28

**Authors:** Yuya Fujita, Manabu Kinoshita, Tomohiko Ozaki, Koji Takano, Kei Kunimasa, Madoka Kimura, Takako Inoue, Motohiro Tamiya, Kazumi Nishino, Toru Kumagai, Haruhiko Kishima, Fumio Imamura

**Affiliations:** 1 Department of Neurosurgery, Osaka International Cancer Institute, Osaka, Japan; 2 Department of Neurosurgery, Osaka University Graduate School of Medicine, Suita, Japan; 3 Department of Neurosurgery, Osaka National Hospital, National Hospital Organization, Osaka, Japan; 4 Department of Thoracic Oncology, Osaka International Cancer Institute, Osaka, Japan

**Keywords:** brain metastasis, EGFR mutation, NSCLC, oligometastasis, prognosis

## Abstract

**Background:**

Molecular and genetic alterations of non-small-cell lung cancer (NSCLC) now play a vital role in patient care of this neoplasm. The authors focused on the impact of epidermal growth factor receptor mutation (EGFR-mt) status on the survival of patients after brain metastases (BMs) from NSCLC. The purpose of the study was to understand the most desirable management of BMs from NSCLC.

**Methods:**

This was a retrospective observational study analyzing 647 patients with NSCLC, including 266 patients with BMs, diagnosed at our institute between January 2008 and December 2015. EGFR mutation status, overall survival (OS) following diagnosis, OS following BMs, duration from diagnosis to BMs, and other factors related to OS and survival after BMs were measured.

**Results:**

Among 647 patients, 252 (38.8%) had EGFR mutations. The rate and frequency of developing BMs were higher in EGFR-mt patients compared with EGFR wildtype (EGFR-wt) patients. EGFR-mt patients showed longer median OS (22 vs 11 months, *P* < .001) and a higher frequency of BMs. Univariate and multivariate analyses revealed that good performance status, presence of EGFR-mt, single BM, and receiving local therapies were significantly associated with favorable prognosis following BM diagnosis. Single metastasis, compared with multiple metastases, exhibited a positive impact on patient survival after BMs in EGFR-mt patients, but not in EGFR-wt NSCLC patients.

**Conclusions:**

Single BM with EGFR-mt performed better than other groups. Furthermore, effective local therapies were recommended to achieve better outcomes.

**Key Points**

- An oligometastatic state exists only in EGFR-mt NSCLC patients.
- BM develops within the initial 2–3 years from the diagnosis of NSCLC.
- Local therapies were effective for survival after BMs.

**Importance of the Study**

The effectiveness of epidermal growth factor receptor tyrosine kinase inhibitors for BMs in EGFR-mt NSCLC patients has dramatically changed treatment strategies and significantly improved overall survival compared with conventional chemotherapy. Furthermore, the concept of oligometastasis has highlighted the importance of local therapies, such as stereotactic radiosurgery and other types of surgery. Using a Cox proportional hazards model, we showed that EGFR-mt NSCLC, single BM, and providing local therapy were associated with significantly longer survival after developing BMs. Single metastasis, compared with multiple metastases, exhibited a positive impact on patient survival after BMs in EGFR-mt patients, but not in EGFR-wt NSCLC patients.

Brain metastases (BMs) are a leading cause of death and imply a poor prognosis. For decades, patients with BMs were often palliatively treated with steroids and whole-brain radiation therapy (WBRT). The discovery of epidermal growth factor receptor tyrosine kinase inhibitors (EGFR-TKIs), however, has dramatically changed treatment strategies for EGFR-mutant non-small-cell lung cancer (NSCLC) patients and significantly improved progression-free survival (PFS) and overall survival (OS) compared with conventional chemotherapy.^1–4^ Likewise, the treatment outcome of BMs from lung cancers has also changed following this paradigm shift. EGFR-TKIs, such as erlotinib and osimertinib, can penetrate the blood–brain barrier^5^ and exhibit a pronounced treatment effect in patients with BMs.^6,7^ Currently, EGFR mutation status is regarded as a key prognostic factor of survival after the diagnosis of BMs.^8^

In addition to this novel paradigm, the concept of oligometastasis, initially proposed by Hellman and Weichselbaum in 1995,^9,10^ has highlighted the importance of local therapies, such as stereotactic radiosurgery (SRS) and other types of surgery.^11^ In patients undergoing surgery before WBRT with a limited number of BMs, the local disease control rate and OS were statistically increased in comparison to patients undergoing only WBRT (20% vs 52% and median 40 vs 15 weeks, respectively). According to 2 randomized controlled studies from Japan and Europe, OS did not differ between SRS alone and a combination of SRS and WBRT. Nowadays, the first choice of treatment for a limited number of BMs is SRS attempting to avoid cognitive impairment due to WBRT. Such treatment achieves favorable survival outcomes with 5-year OS, reaching 29.4% for patients with oligometastatic NSCLC.^12^ With improved survival outcomes of patients with BMs, it is now more important to understand the characteristics of NSCLC patients with BMs. The purpose of this study was to clarify the influence of EGFR mutation status and local therapies on the survival of NSCLC patients with BMs and to elucidate prognostic factors for the patient population.

## Materials and Methods

### Patients

This study was reviewed and approved by the Research Ethics Committee of Osaka International Cancer Institute (approval number: 1707109126). Written informed consent was obtained from all patients. All data were fully anonymized, and the protocol was conducted in agreement with the Declaration of Helsinki.

We retrospectively investigated patients diagnosed with NSCLC at our institute between January 2008 and December 2015. The inclusion criteria for this study were defined as follows: patients diagnosed with NSCLC, available EGFR mutation status, available computed tomography (CT) or magnetic resonance imaging (MRI) of the brain, and an observational period of 2 years or more. Among 676 NSCLC patients, we excluded 26 for lacking EGFR mutation testing and 3 for insufficient clinical data, such as CT or MRI, leaving a cohort of 647 patients in total for analysis.

The analysis was performed in 2 stages. First, we analyzed all 647 patients diagnosed with NSCLC to investigate temporal patterns in the occurrence of BMs. In the second stage, 266 patients who developed BMs were analyzed to reveal the prognostic impact of EGFR mutation status and treatment.

EGFR mutation testing was performed at LSI Medience Corporation using a peptide nucleic acid-locked nucleic acid PCR clamp method.^13^ The number of BMs was counted manually on contrast-enhanced CT or MRI performed at the time when BMs were initially identified.

### Statistical Analysis

Statistical analysis was performed using JMP Pro version 14 (SAS Institute, Inc.). Fisher’s exact test for categorical variables was used to compare patient and disease characteristics according to EGFR mutation status. The Kaplan–Meier method using the log-rank test was used to analyze OS and survival duration after BMs. A Cox proportional hazards model was used to analyze the risk factors of developing BMs. A *P*-value of <0.05 was considered statistically significant.

## Results

### Demography of Patients With NSCLC

Table 1 presents the characteristics of 647 patients with NSCLC and 266 patients with NSCLC who developed BMs. There were 252 patients with EGFR-mutant (EGFR-mt) (38.8%) and 395 patients with EGFR-wild type (EGFR-wt) (61.1%) NSCLC. Nearly half of EGFR-mt NSCLC patients had an exon 19 deletion (50.7%), and most others had an L858R point mutation (47.6%). Of the 266 patients who developed BMs, there were 127 EGFR-mt (47.7%) and 139 EGFR-wt (52.3%). Histopathological specimens mainly showed adenocarcinoma (87.2%), followed by squamous cell carcinoma (9.3%). Treatment modalities for BMs from EGFR-mt NSCLC patients were comprised of EGFR-TKI (76.4%), surgical resection (6.3%), SRS (53.5%), WBRT (30.0%), and immune checkpoint inhibitors (ICIs) (4.0%). EGFR-mt NSCLC patients were administered with the following EGFR-TKIs: gefitinib (54.3%), erlotinib (62.2%), afatinib (17.3%), and osimertinib (8.7%). Treatment modalities for BMs from EGFR-wt patients were mainly SRS (59.7%), followed by WBRT (38.8%), surgical resection (13.7%), and ICIs (7.1%). BMs from EGFR-wt NSCLC were significantly more frequently surgically resected than those from EGFR-mt NSCLC patients (*P* = .047, Fisher’s exact test).

**Table 1.** The Characteristics of 647 Patients With NSCLC and 266 Patients With NSCLC Who Developed BMs

Patient Characteristics				
NSCLC	EGFR mutated (*n* = 252)	EGFR wildtype (*n* = 395)	All (*N* = 647)	
	*N* (%)	*N* (%)	*N*	*P*
Age at diagnosis, years				
Median	63.8 ± 9.7	64.1 ± 10.3	64.0 ± 10.1	
Female	108 (42.9)	136 (27.5)		
Male	144 (57.1)	259 (72.5)		
EGFR mutation				
Ex19Del	128 (50.7)			
L858R	120 (47.6)			
Uncommon mutation	4 (1.70)			
NSCLC with BM	EGFR mutated (*n* = 127)	EGFR wildtype (*n* = 139)	All (*N* = 266)	
	*N* (%)	*N* (%)	*N*	*P*
Age at diagnosis, years				
Median	63.0 ± 9.98	60.9 ± 10.4	61.9 ± 10.2	
Female	75 (60.4)	49 (39.5)	124	
Male	52 (36.6)	90 (63.4)	142	<.001
Stage at diagnosis				
I–III	36 (50.7)	35 (49.3)	71	
IV	91 (46.7)	104 (53.3)	195	
ECOG performance status				
0–1	108	123	231	
2–4	18	16	34	
Number of brain metastases				
1–3	37	50	87	
4–20	23	19	42	
>20	5	5	10	
Tissue type				
Adenocarcinoma	122	110	232	
Squamous cell carcinoma	4	21	25	
Large cell neuroendocrine carcinoma	0	2	2	
Pleomorphic cell carcinoma	0	5	5	
NSCLC (unclassified)	1	1	2	
EGFR mutation				
Ex19Del	57 (44.9)			
L858R	67 (52.8)			
Uncommon mutation	3 (0.24)			
Treatment for BM by				
EGFR-TKI	96 (75.6)			
Gefitinib	69 (54.3)			
Erlotinib	79 (62.2)			
Afatinib	22 (17.3)			
Osimertinib	11 (8.7)			
Operation	8 (6.3)	19 (13.7)	27	.047
SRS	68 (53.5)	83 (59.7)	151	
WBRT	38 (30.0)	54 (38.8)	92	
ICI	5 (4.0)	10 (7.1)	15	

EGFR, epidermal growth factor receptor; ECOG, Eastern Cooperative Oncology Group; Ex19del, exon 19 deletion; TKIs, tyrosine kinase inhibitors; SRS, stereotactic radiosurgery; WBRT, whole-brain radiation therapy; ICIs, immune checkpoint inhibitors; BMs, brain metastases.

### Rate and Frequency of Developing BMs in NSCLC

**Figure 1.**
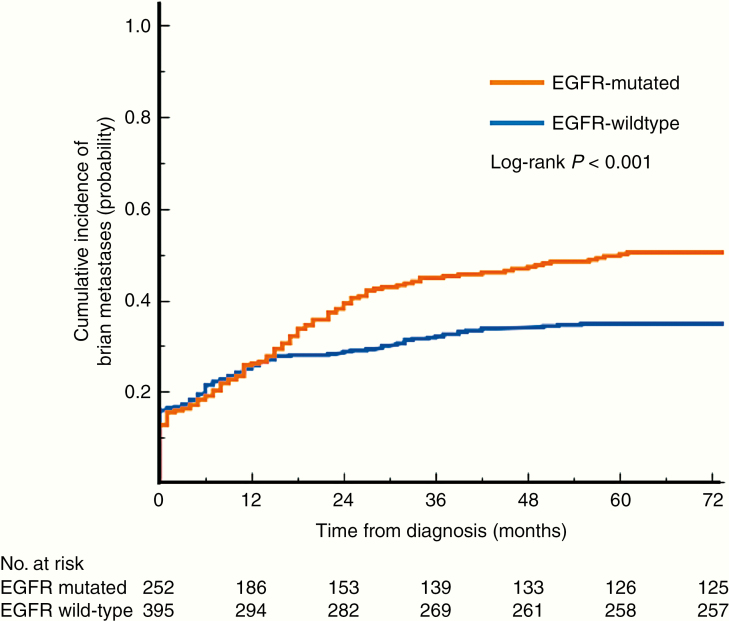
Cumulative incidence of BMs. EGFR-mt patients developed BMs more quickly, and the rate of BMs reached about 50%, while the rate for EGFR-wt patients was about 30% (*P* < .001, log-rank test). Almost 80% of patients developed BMs within 2–3 years following the diagnosis of NSCLC. EGFR, epidermal growth factor receptor; mt, mutated; wt, wild type; BMs, brain metastases.

Figure 1 shows the cumulative incidence of BMs from NSCLC grouped by EGFR mutation status. The rate of EGFR-mt NSCLC patients harboring BMs at the initial presentation was lower than EGFR-wt patients (12.7% vs 15.9%). It should be noted, however, that EGFR-mt NSCLC patients developed BMs more rapidly than EGFR-wt patients and that the cumulative rate of developing BMs reached close to 50% in this population. Meanwhile, the cumulative percentage of EGFR-wt patients developing BMs was approximately 30% (*P* = .0006, log-rank test). BMs mostly developed within 2–3 years after the initial diagnosis of NSCLC.

### Treatment Outcome After Developing BM in NSCLC

**Figure 2.**
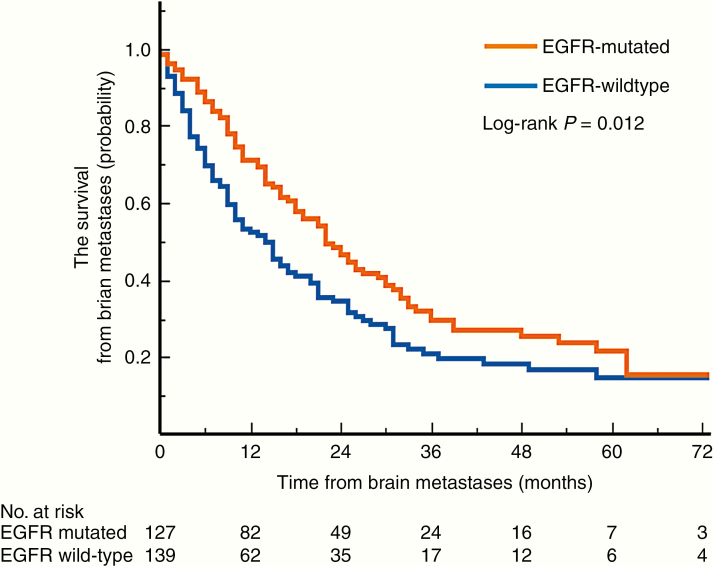
A Kaplan–Meier curve illustrating the survival of EGFR-mt and EGFR-wt NSCLC patients after BMs (22 vs 11 months, *P* = .012, log-rank test). EGFR, epidermal growth factor receptor; mt, mutated; wt, wild type; BMs, brain metastases.

Concerning clinical courses after developing BMs, EGFR-mt NSCLC patients exhibited longer median OS than EGFR-wt patients. Still, the long-term survival rate did not differ between the 2 groups, as shown in Figure 2 (median survival 22 vs 11 months, *P* = .012, log-rank test). In the sub-analysis, there was no difference in the duration of survival after developing BMs between those who had exon 19 deletion and exon 21 L858R mutation (median survival 22 vs 24 months, *P* = .35, log-rank test). The administration of third-generation EGFR-TKIs was associated with longer survival after developing BMs, compared with first-generation EGFR-TKIs (median survival unreached vs 19 months, *P* < .001, log-rank test). EGFR-mt NSCLC (hazard ratio [HR] 0.62; confidence interval [CI] 0.39–0.98; *P* = .041), single BM (HR 0.61; CI 0.43–0.87; *P* = .0057), and local therapy (HR 0.60; CI 0.65–1.67; *P* = .0002) were associated with significantly longer survival after developing BMs, whereas poor performance status (HR 2.02; CI 1.22–3.21; *P* = .008) was significantly associated with poorer prognosis both in univariate and multivariate analyses (Table 2). Sex (HR 0.72; CI 0.51–1.00; *P* = .050) and patient age older than 65 years (HR 0.78; CI 0.55–1.12; *P* = .17) did not correlate with prognosis. WBRT was significantly associated with poorer prognosis in univariate analysis (HR 1.56; CI 1.16–2.10; *P* = .0037), which did not hold to be statistically significant in multivariate analysis (HR 1.26; CI 0.33–1.46; *P* = .21).

**Table 2.** Univariate and Multivariate Analyses for Overall Survival After BM

	Univariate analysis			Multivariate analysis		
Variables	*P*	Hazard ratio	95% CI	*P*	Hazard ratio	95% CI
Female vs male	.05	0.75	1.00–1.78	.05	0.72	0.51–1.00
Age <66 vs ≧66 years	.05	0.74	0.55–1.00	.17	0.78	0.55–1.12
ECOG PS 2–3 vs 0–1	<.0001	2.89	1.85–4.35	.008	2.02	1.22–3.21
EGFR mutated vs wildtype	.01	0.69	0.52–0.93	.032	0.61	0.39–0.96
Single BM	.0006	0.59	0.43–0.80	.0057	0.61	0.43–0.87
The use of EGFR-TKI	.94	0.99	0.72–1.35	.6	1.13	0.72–1.79
Local therapies (SRS, operation)	<.0001	0.49	0.37–0.67	.0002	0.51	0.36–0.72
WBRT	.0037	1.56	1.16–2.10	.21	1.26	0.88–1.80
Use of ICIs	.33	0.71	0.32–1.36	.42	0.75	0.33–1.46

The hazard ratio was estimated in a Cox proportional hazard model.

BMs, brain metastases; ECOG PS, Eastern Cooperative Oncology Group Performance Status; EGFR, epidermal growth factor receptor; TKIs, tyrosine kinase inhibitors; SRS, stereotactic radiosurgery; WBRT, whole-brain radiation therapy; ICIs, immune checkpoint inhibitors.

**Figure 3.**
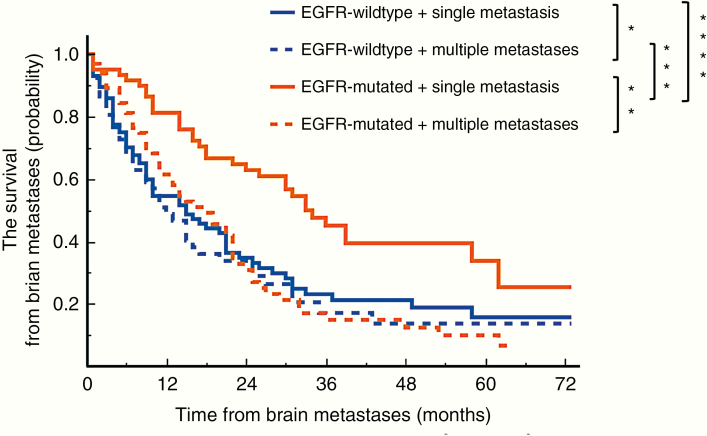
This figure shows the Kaplan–Meier curve of EGFR mutation status and the number of BMs (single vs multiple). The group of EGFR-mt patients with a single metastasis exhibited significantly longer survival after BM occurrence (****P = .0061 vs EGFR-wt patients with single BM, ***P = .0013 vs EGFR-wt patients with multiple BMs, **P = .0009 vs EGFR-mt patients with multiple BMs). There was no significant difference between single and multiple metastases in EGFR-wt patients (*P = 1.00). All data are reported as an adjusted *P*-value, log-rank test with Bonferroni correction. EGFR, epidermal growth factor receptor; mt, mutated; wt, wild type; OS, overall survival; BMs, brain metastases.

A comparison between single and multiple metastases is shown in Figure 3. For BMs in EGFR-mt patients, single metastasis, compared with multiple metastases, exhibited a positive impact on patient survival (*P* = .0009, log-rank test with Bonferroni correction) but not in EGFR-wt NSCLC patients (*P* = 1.00, log-rank test with Bonferroni correction). Furthermore, there was no statistical difference between EGFR-mt and EGFR-wt patients with multiple BMs. Median survival after BMs was 33 months in EGFR-mt NSCLC patients with a single BM and 18 months for patients with multiple BMs. On the other hand, the median survival after BMs was 18 months in EGFR-wt NSCLC patients with a single BM and 12 months for patients with multiple BMs. The type of EGFR mutations did not have any impact on survival after BMs neither in cases of single nor multiple BMs (*P* = 1.00 and *P* = 1.00, log-rank test with Bonferroni correction). Caution should be taken, however, when interpreting this result, as it is possible that the result is heavily impacted by selection bias and the retrospective nature of the study.

## Discussion

Recently, combination therapy, including surgery, SRS, EGFR-TKI, ICI, and WBRT, has significantly improved the prognosis of NSCLC. This improvement has thrown into question the impact of BMs on NSCLC, especially concerning EGFR mutation status. In an attempt to answer this question, the current study succeeded in providing 3 insights.

First, the number of BMs exhibited different effects on OS in EGFR-mt and EGFR-wt NSCLC patients. This finding verifies the findings of Yuan et al.^14^ In their report, Yuan et al. analyzed the duration from the initial diagnosis to BM development and divided the cohort into “early” (less than 6 months from initial diagnosis) and “late” (more than 6 months from diagnosis) groups. Median OS from diagnosis was 7.1 months in the “early” group and 24.9 months in the “late” group for EGFR-wt patients. On the other hand, there was no statistical difference between the “early” group and “late” group for EGFR-mt patients. Likewise, the median OS from BMs for EGFR-wt patients was not significantly different between the early (6.3 months) and the late groups (4.9 months). This is despite a statistical difference between early (19.2 months) and late groups (3.9 months, *P* < .001) for EGFR-mt patients.^14^ Although the cause of this difference remains undetermined, it may be due to the fact that EGFR-wt NSCLC patients develop BMs in the final stages of the disease. In contrast, EGFR-mt NSCLC patients may develop BMs even at early stages when the disease is still responsive to therapy. In other words, as for NSCLC patients with BMs, the oligometastatic state exists only in EGFR-mt NSCLC patients.

The second significant observation was the temporal pattern of BM development in NSCLC patients. A previous study reported that there was a higher incidence of BMs in patients with EGFR-mt NSCLC, and most BMs occurred within 3 years from the initial diagnosis of NSCLC regardless of EGFR mutation status.^15^ Consistent with this report, our findings underscore the prevalence of BM development during the first 2–3 years following the diagnosis of NSCLC.

Finally, we confirmed the importance of local therapies for BMs from NSCLC. Our analysis suggested that local treatments, such as surgical resection and SRS, were significantly associated with prolonged survival after developing BMs, while the use of EGFR-TKI, ICI, and WBRT was not. Chao et al.^16^ similarly reported that SRS was able to achieve local control. On the other hand, a randomized clinical trial showed that there was no significant difference between patients receiving WBRT and patients receiving no further treatment,^17^ which is similar to the findings of this study. Although the use of EGFR-TKI for BMs implies a certain level of efficacy,^6,7^ the efficacy of upfront EGFR-TKI preceding SRS or WBRT for BMs is still controversial.^18,19^ Furthermore, a recent study suggested that OS and PFS are possibly extended by the combination of EGFR-TKI and cytotoxic chemotherapy.^20^

The limitations of this study should be considered. First, the cohort in this study mainly consisted of Japanese nationals. A Japanese-oriented cohort could have biased results, as the race is known to be a critical factor when trying to understand NSCLC. For example, Asian patients more frequently present with EGFR-mt NSCLC (34–67%) than patients in other regions (8–26%).^21^ It is also known that the use of EGFR-TKI causes interstitial pneumonia more often in Asian populations.^22^

Nonetheless, the rate and frequency of developing BMs in this study were not different from those of previous reports.^15,23^ Secondly, it should be cautioned that the occurrence of BMs was possibly underrepresented in this study. The guidelines of the Japan Lung Cancer Society recommend checking for the presence of BMs at diagnosis with gadolinium-enhanced MRI only when the primary tumor size in the lungs is larger than 2 cm. Otherwise, it is not recommended to check for the presence of BMs. This study adhered to these guidelines, leaving open the possibility that BMs were not screened for in cases with small-size tumors. Finally, the choice of treatment for BMs could have been biased in this study due to a cohort selected by a single cancer-treating institution. A prospective study is needed to address these concerns.

In conclusion, the current study suggests that NSCLC patients should be followed up carefully within the initial 2–3 years following diagnosis to monitor for the occurrence of BMs. Furthermore, if BMs develop, the current study suggests that patients with EGFR-mt NSCLC with single BM have a favorable prognosis, and local curative therapies should be considered.

## Funding

This research was funded by the Japan Society for the Promotion of Science (16K10778 and 17H05308); Takeda Science Foundation; and MSD Life Science Foundation.

*Conflict of interest statement*. The authors report no conflict of interest.

## Authorship Statement

Y.F., M.K., and F.I. conceived and designed the analysis. Y.F., M.K., K.T., K.K., M.K., T.I., M.T., K.N., T.K., and F.I. acquired the data. Y.F. conducted statistical analysis under the supervision of M.K., H.K., and F.I. All authors helped in interpreting the findings. Y.F. wrote the first draft of the manuscript. All authors contributed toward subsequent revisions and approved the submitted manuscript.
